# Neurovascular Unit as a Source of Ischemic Stroke Biomarkers—Limitations of Experimental Studies and Perspectives for Clinical Application

**DOI:** 10.1007/s12975-019-00744-5

**Published:** 2019-11-07

**Authors:** Aleksandra Steliga, Przemysław Kowiański, Ewelina Czuba, Monika Waśkow, Janusz Moryś, Grażyna Lietzau

**Affiliations:** 1 Faculty of Health Sciences, Pomeranian University of Slupsk, 64 Bohaterów Westerplatte St., 76-200 Slupsk, Poland; 2 Department of Anatomy and Neurobiology, Medical University of Gdansk, 1 Debinki St., 80-211 Gdansk, Poland; 3 Department of Clinical Science and Education, Södersjukhuset, Internal Medicine, Karolinska Institutet, Stockholm, Sweden

**Keywords:** Astrocytes, Biomarkers, Neuroglia, Neurovascular unit, Stroke

## Abstract

Cerebral stroke, which is one of the most frequent causes of mortality and leading cause of disability in developed countries, often leads to devastating and irreversible brain damage. Neurological and neuroradiological diagnosis of stroke, especially in its acute phase, is frequently uncertain or inconclusive. This results in difficulties in identification of patients with poor prognosis or being at high risk for complications. It also makes difficult identification of these stroke patients who could benefit from more aggressive therapies. In contrary to the cardiovascular disease, no single biomarker is available for the ischemic stroke, addressing the abovementioned issues. This justifies the need for identifying of effective diagnostic measures characterized by high specificity and sensitivity. One of the promising avenues in this area is studies on the panels of biomarkers characteristic for processes which occur in different types and phases of ischemic stroke and represent all morphological constituents of the brains’ neurovascular unit (NVU). In this review, we present the current state of knowledge concerning already-used or potentially applicable biomarkers of the ischemic stroke. We also discuss the perspectives for identification of biomarkers representative for different types and phases of the ischemic stroke, as well as for different constituents of NVU, which concentration levels correlate with extent of brain damage and patients’ neurological status. Finally, a critical analysis of perspectives on further improvement of the ischemic stroke diagnosis is presented.

## Introduction

Despite constantly increasing understanding of ischemic stroke pathophysiology, resulting among others from laboratory and clinical studies, improvement of neuroradiological imaging technics, better understanding of the significance of risk factors and application to clinical practice the results of large population studies, diagnosis of acute stroke is frequently very difficult. An improvement of the organization of stroke centers and emergency transport has resulted in a significant reduction of door-to-needle time. However, despite the efforts made, this does not concern all countries and all stroke centers. In fact, a significant proportion of stroke patients still arrive to the hospital and are diagnosed late or even too late to start thrombolytic therapy [1]. The problem of the effective, fast, and precise diagnosis remains a serious challenge. In addition, as confirmed by numerous publications, in the earliest period of ischemic stroke, its diagnosis, even applying the most advanced neuroradiological methods, is frequently difficult or impossible. The results of magnetic resonanse imaging (MRI) and computer tomography (CT) studies conducted within 24 h from the symptoms onset even by the experienced neuroradiologists are often inconclusive. Inability to precisely diagnose a patient in the early phase of ischemic stroke may reach even 30% of cases [2]. Despite the advances in organization of stroke treatment, there is a number of cases that can create diagnostic difficulties [3, 4]. This can result from lack of the characteristic symptoms (also stroke mimics), assessment by non-specialists (at the pre-hospital phase or during transport), normal CT image, inaccessibility of neuroradiological equipment such as MRI or angiography, and, finally, in cases where documentation of the disease is incomplete (patient’s history is missing, onset of symptoms is unknown, clinical examination is incomplete). In such cases, use of diagnostic tests based on biomarkers’ panels would be rationally justified. Thus, this creates a gap for introduction of simple, not expensive, and reliable diagnostic methods, including these based on use of the neurovascular unit (NVU)-derived biomarkers, being in a focus of this review. Taking into account 4.5-h length of the therapeutic window, accepted for introduction of the thrombolytic therapy, fast and reliable diagnosis of ischemic stroke has unquestionable practical significance. The gold standard and the goal of searching for the optimal biomarker of an ischemic infarct is a sensitive substance that is objectively measured and evaluated as an indicator of physiological and pathological processes, which is specific for a given type of stroke, which content increases during a specific period of time after the symptoms’ onset and reflects responses to therapeutic interventions [5]. The biomarker’s value is also determined by its availability for diagnostic purposes, being a consequence of its presence in physiological fluids, such as blood or cerebrospinal fluid (CSF). Hence, our work focused on reviewing already-used, and also potential, indicators of ischemic stroke present primarily in blood serum and CSF. In addition, an attention was also paid to changes in the content of potential markers in the nervous tissue, which correlate with intensity of brain edema, assessed in neuroradiological evaluation based on MRI or positron emission tomogrphy (PET) imaging (e.g., aquaporin-4; AQP4). Despite intensive studies, so far it has not been possible to identify a single biomarker or a set of markers that would meet the expected criteria of sensitivity and specificity for detection of different types of stroke (e.g., cardioembolic, lacunar, thrombotic or large-vessel infarct, etc.) or could give a chance to predict the severity of neurological deficits, and complications or benefits resulting from different forms of implemented treatment [4, 6, 7]. All these difficulties are, at least in part, a consequence of the complex morphological structure of brain tissue and the plethora of pathophysiological processes triggered in the course of ischemic stroke [3]. Another important factor that could be responsible for failure in searching for effective ischemic biomarkers is lack of commonly accepted standardization and analytical validation of laboratory procedures, making difficult comparisons between the results obtained in different laboratories [8]. Hence, unlike the myocardial infarction, the advantages of using biochemical markers of the cerebral stroke are much more limited. Due to the fact that previously published results on potential ischemic biomarkers are inconclusive, we think it would be of some value to change the approach to this research problem and adopt some new assumptions in selection of potential markers of the ischemic process. Considering the complex structure of brain tissue and dynamic nature of processes occurring in the course of ischemia, two following issues should be taken into account during selection of markers with potential diagnostic significance: (1) the contribution of all morphological components of brain tissue to secretion of potential biomarkers; (2) the dynamic nature of pathophysiological processes occurring in the course of cerebral stroke, with their assignment to its acute, subacute, and late phases. An alternative approach is especially required for elaboration of reliable and effective diagnostic measures for stroke recognition in its acute phase. In order to improve the diagnostic effectiveness, it seems to be justified to validate the concept of NVU in the further research. The NVU has proved its unquestionable value for explanation of the mechanisms of several physiological and pathological processes in the CNS [9–11]. This morphological and functional conceptual model of the nervous tissue enables easier choice of several potential biomarkers useful for stroke evaluation and prognosis. Hence, further research should comprise the diagnostic panels, consisting of biomarkers representative not only for neurons but also for neuroglia, extracellular matrix, and elements of the blood–brain barrier (BBB) [12, 13]. Three main components of the NVU, namely neurons, neuroglia, and brain vessels, reveal different resistance to ischemic conditions and different involvement in metabolic processes and play a different role in immune response to pathological processes occurring in the brain (e.g., cell death, brain edema, reactive gliosis, or BBB disintegration) [14]. Activation of NVU during pathological processes induces release of numerous potential biomarkers into the extracellular space, blood vessels, and CSF. Some of them are characteristic for definite cellular components, such as neurons or astrocytes, e.g., neuron-specific enolase (NSE) or glial fibrillary acidic protein (GFAP), respectively, whereas the others are released by several of them, e.g., cytokines or neurotrophic factors [8]. Altogether, development of diagnostic panels consisting of biomarkers representing all elements of the NVU and revealing the highest specificity for different phases of stroke could contribute to significant improvement of stroke diagnosis.

## Elements of the Neurovascular Unit in Pathophysiology of Brain Ischemia

**Fig. 1:**
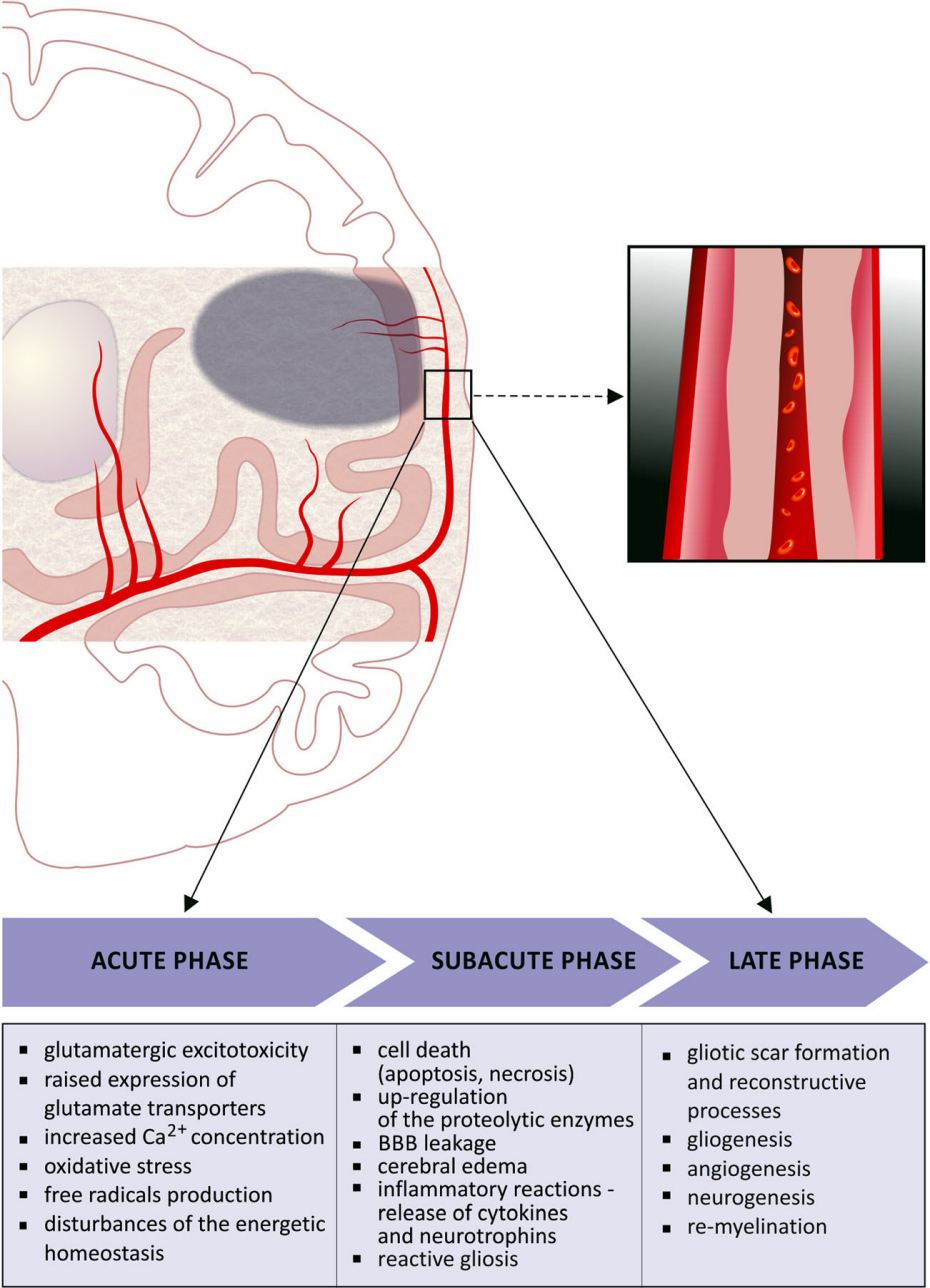
Summary of pathophysiological processes developing in the course of ischemic stroke. The consequences of ischemic stroke result from processes beginning in a strictly defined time sequence. A decrease in cerebral blood flow results in reduction of oxygen and glucose delivered to the brain tissue. This initiates a cascade of biochemical processes ultimately leading to cellular destruction and death. One of the earliest consequences of these processes is change of oxidative glycolysis into less effective anaerobic pathway, followed by decrease in ATP production and raising lactate concentration. This results in decrease in all energy-dependent metabolic processes and dysfunction of the ion pumps leading to changes of ion concentrations (i.e., decrease in K^+^ intracellular levels and increase in Cl^−^, Na^+^, and Ca^2+^ levels). This in turn contributes to influx of water and development of brain swelling. Depolarization of neuronal cell membranes leads to release of excessive amounts of glutamate and triggering glutamatergic excitotoxicity. Stimulating effect of glutamate, by changing Ca^2+^ concentration and activation of enzymes (e.g., proteases, lipases, phosphatases, and endonucleases), leads to further cell destruction. This is accompanied by the activation of oxidative stress along with an increase in production of free oxygen and nitrogen radicals. A further consequence of these processes is triggering of inflammatory response and release of proinflammatory cytokines and chemokines which destructive action affects all elements of neurovascular unit (NVU). In cerebral vessels, increase in permeability of BBB and damage to the endothelial cells occur, accompanied by up-regulation of thrombotic mechanisms. These processes are accompanied by apoptotic and necrotic cell death, which are dependent on the length of cerebral blood flow reduction, extent of the energetic metabolism disturbances, and localization of the cells (within infarct core or penumbra). Decrease in the cerebral blood flow and reduced availability of oxygen and glucose initiate the *acute phase* (up to 24 h from ischemia onset) processes in the course of ischemic stroke, i.e., glutamatergic excitotoxicity, increase in Ca^2+^ levels, and anaerobic glycolysis, leading to reduced efficiency of the energetic metabolism. The *subacute phase* (up to 7 days from ischemia onset) is characterized by occurrence of various forms of cell death, the blood–brain barrier (BBB) disintegration and leakage, as well as, initiation of the inflammatory response followed by release of mediators exacerbating the effects of primary damage. In contrast to the earlier phases, during the *late phase* (starting one week from ischemia onset), the initiated processes lead to limiting of deleterious effects of the cerebral blood flow reduction through development of reactive gliosis and gliotic scar, which enables demarcation of the necrotic infarct core from the surrounding intact tissue. At this phase, the reparative processes resulting from cell proliferation and differentiation dominate, what is reflected in intensive reconstruction of the cellular populations, angiogenesis and re-myelination.

Deleterious effects of cerebral ischemia result from coincidence of several factors, such as time length and depth of cerebral blood flow decrease, permanent or transient character of ischemia and its localization [15, 16]. Some anatomical features, like density of cerebral capillary vasculature or presence of collateral circulation, can also contribute to the final outcome [17]. Among consequences of ischemic stroke, developing within 24 h (acute phase) from ischemia onset, the following should be encountered: the glutamatergic excitotoxicity and raised expression of glutamate transporters [18], increased Ca^2+^ ion concentration, and oxidative stress, accompanied by free radical production (Fig. 1) [19, 20]. Apoptosis and necrosis can occur within the first few hours after ischemia onset and reach their peaks after 24 h [21]. During that time, upregulation of the apoptotic and necrotic markers and proteolytic enzymes (e.g., metalloproteinases; MMPs) occurs [22]. The BBB damage is followed by activation of the endothelial cells and pericytes [23, 24]. This is related with release of some specific markers, such as von Willebrand factor (vWF) and nerve growth factor (NGF) [25, 26]. The BBB leakage leads to development of cerebral edema and changes in AQP4 expression in astrocytes, with its peak at 3–4 days (subacute phase) after the ischemia onset [27, 28]. The cerebral edema subsides gradually, followed by the AQP4 concentration returning to the normal level after 1 week (late phase). Activation of the inflammatory response is signalized by release of many mediators, such as cytokines and neurotrophins, predominantly non-specific for a single cellular population and produced by several constituents of the NVU. The reactive gliosis could be encountered among the subacute and late-phase phenomena [29]. It is related with neuroglial proliferation and hypertrophy, all of which are signalized by production of characteristic markers, e.g., GFAP or S100 beta (S100β) protein. In all these processes, astrocytes play a unique role resulting from their resistance to the reduced glucose and oxygen supply, anaerobic glycolysis, and lactate production (i.e., astrocytic-neuronal lactate shuttle), as well as resulting from the homeostatic function and immunological competence [30, 31]. Finally, brain ischemia triggers changes in expression of genes, which are involved in regulation of the structural proteins and inflammatory mediators, as well as transcription and neurotrophic factors [32, 33]. Acidosis, resulting from increased lactate level in the course of anaerobic glycolysis, as well as increased proton concentration, produced by ATP hydrolysis, is associated with activation of acid-sensing ion channels (ASICs). The increased influx of Ca^2+^ ions into the neurons augments the destructive Ca^2+^-dependent processes in the pathway which is independent from activation of glutamate receptors [34]. This mechanism has been proved to intensify neuronal damage in the course of ischemia.

As it could be inferred from this brief summary, a search for the potential ischemic biomarkers should be concentrated not only on indicators representative for neurons, but also neuroglial and endothelial cells, expressed in the acute and later phases of cerebral stroke, and activated in the course of different processes. This attitude could give a chance to screen possibly the largest spectrum of pathological processes, represented by selected markers, and provide a comprehensive information, concerning various aspects of ischemic stroke.

## Choice of Potential Candidate Ischemic Stroke Biomarkers

**Fig. 2:**
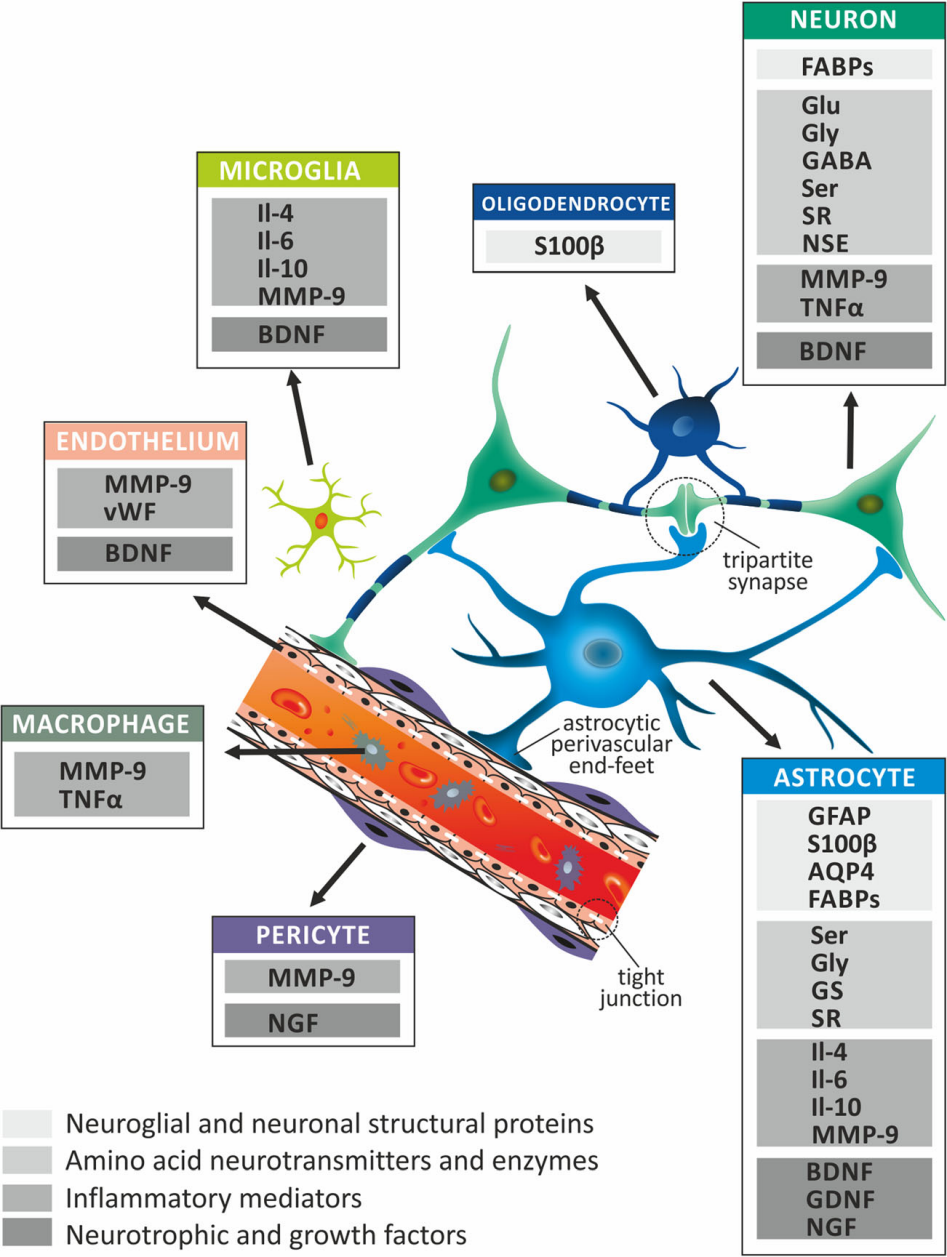
The neurovascular unit (NVU) as a source of ischemic stroke biomarkers. The NVU concept has been proved useful for analysis of spatial and functional relationships among constituents of brain tissue. An important role of NVU is attributed to its characteristic morphological structures, such as tripartite synapses, astrocytic perivascular end-feet and vascular tight junctions. In accordance with the NVU concept, ischemic stroke biomarkers can be categorized as the representatives of either individual cell type or its several components. In addition, this concept enables division of ischemic stroke biomarkers into groups characterized by similar structure and functions representing the following categories: (1) neuroglial and neuronal structural proteins, (2) amino acid neurotransmitters and enzymes, (3) inflammatory mediators, and (4) neurotrophic and growth factors. While planning the research on the new ischemic stroke biomarkers, it is important to take into account that various components of NVU play different roles in the ischemic metabolic processes (e.g., oxidative and anaerobic glycolysis); signaling pathways (e.g., glutamate-glutamine shuttle, Ca^2+^ ion- and purines-based signaling) reveal different sensitivity to decreased cerebral blood perfusion, as well as, reveal different proliferation potential. Consequently, the NVU concept is useful for assessment of brain tissue damage, which is reflected in concentration changes of various NVU-derived biomarkers that translocate from the brain to blood and CSF. AQP4, aquaporin-4; BDNF, brain-derived neurotrophic factor; FABPs, fatty acid–binding proteins; GABA, γ-aminobutyric acid; GDNF, glial cell line–derived neurotrophic factor; GFAP, glial fibrillary acidic protein; Glu, glutamate; Gly, glycine; GS, glutamine synthetase; Il-4, interleukin-4; Il-6, interleukin-6; Il-10, interleukin-10; MMP-9, matrix metalloproteinase-9; NGF, nerve growth factor; NSE, neuron-specific enolase; S100β, S100beta protein; Ser, serine; SR, serine racemase; TNFα, tumor necrosis factor α; vWF, von Willebrand factor

Taking into consideration all the abovementioned issues related with the concept of NVU, a selection of the ischemic stroke biomarkers has been chosen and reviewed (Fig. 2). We focused on reviewing the most important, recognized, and potentially useful substances that can serve as biomarkers of the NVU lesion, as well as indicators of processes related to the functioning of this unit. The relationship between NVU and stroke biomarkers is not limited to the fact that specific markers are produced by different types of NVU cells. It is closely related to the integrated function of all elements of the NVU in regulation of the cerebral blood flow, BBB integrity, and permeability, as well as energetic metabolism and immunological response. Disintegration of this complicated system leads to upregulation of different biomarkers and enables their passage into blood vessels. The result being a specific profile of the biomarkers’ content in the blood and CSF. This reflects the severity of tissue damage and intensity and nature of blood flow disturbance (permanent or transient) and provides information shedding light on stroke etiopathogenesis (occurrence of particular set of biomarkers). Thus, we have just started to learn the complex pathophysiological relationships within NVU, related to the production and release of ischemic stroke biomarkers. Many previous articles discuss in detail properties of the substances that are already used, or could potentially be used in the future, as new ischemic stroke biomarkers. However, a significant proportion of them are non-specific for brain tissue and are produced outside of the CNS, as a manifestation of a systemic response to brain damage, caused by decrease in blood flow, BBB damage, oxidative stress, or initiation of inflammatory processes. In this review, we have focused on biomarkers produced by components of the nervous tissue that are involved in pathophysiological processes and morphological changes occurring in the brain. In summary, this review article emphasizes the morphological and functional aspects of the relationship between NVU components, both under physiological conditions and in the course of ischemia, which result in expression changes and release of specific biomarkers from the brain tissue into the vascular system and/or CSF.

This review contains only some representatives of the extensive list of potential candidate substances (Table 1). Following, we discuss those of them which are already used in clinical practice and have a documented diagnostic significance, as well as those revealing potential usefulness, based on preliminary results of in vitro and in vivo studies, carried out in animal models. It is worth mentioning that due to the complex etiopathogenesis of ischemic stroke and dynamic nature of processes occurring in its different types, the analysis of diagnostic applicability of particular biomarkers requires thorough understanding of their role and knowledge about the changes in their expression in each type and phase of ischemic infarct. In the case of many potential biomarkers, the results of such studies are not available yet. Moreover, while choosing the potential markers for further clinical trials, the results of studies obtained exclusively based on in vitro or in vivo protocols, carried out on the representatives of single species of laboratory animals, should be treated with caution, as it is highly probable to expect significant interspecies differences in regulatory mechanisms, as well as differences in expression of particular markers resulting from the evolutionary and phylogenetic reasons [130].

**Table 1:** Ischemic stroke biomarkers, summary of function, and diagnostic value

Marker		Localization	Function and diagnostic significance	References
Neuroglial and neuronal structural proteins				
Glial fibrillary acidic protein	GFAP	Astrocytes	Marker of cellular integrity and reactive gliosis, upregulated in ischemic and hemorrhagic stroke and also in traumatic brain injury; useful for differentiating between ischemic and hemorrhagic stroke; correlation reported between GFAP concentration in CSF and neurological status	[29, 35–39]
S100β protein	S100β	Astrocytes, oligodendrocytes and Schwann cells	Marker of neuroprotective or neurotoxic function depending on its serum concentration, supports neurogenesis and neuronal differentiation, in higher concentrations promotes necrotic and apoptotic cell death; sensitive biomarker of ischemic stroke, valuable for assessment of stroke malignant and hemorrhagic transformation and for stroke mimics exclusion; correlation reported between S100β serum and CSF level and infarct volume	[38, 40–45]
Aquaporin-4	AQP4	Astrocytic perivascular end-foot	AQP4 facilitates astrocytic swelling in cytotoxic edema and enhances water clearance in vasogenic edema; revealed association between AQP4 expression and intensity of brain edema studied in MRI and PET; potential biomarker of brain edema and status of physiological brain barriers	[46–49]
Fatty acid–binding proteins	FABPs	Neurons and neuroglia	Proteins responsible for maintaining of appropriate serum concentration of the long-chain fatty acids; potentially valuable for assessment of infarct volume, prediction of neurological deficits and differentiation among types of ischemic infarcts	[50–53]
Amino acid neurotransmitters and enzymes				
Glutamate	Glu	Neurons, astrocytes	Widely distributed excitatory neurotransmitter; valuable for assessment of specific types of stroke; changes of its concentration in blood serum and CSF correlate with progressive or stable dynamics of ischemic stroke, changes of neurological status, early deterioration and infarct volume	[54–56]
Glycine	Gly	Neurons, astrocytes	Amino acid neurotransmitter regulating neuronal activity; changes of its serum or CSF concentration can be related with development of functional deficits or excitatory symptoms; diagnostic value of Gly for prediction of outcome in different types of stroke and differentiation ischemic stroke from negative control requires further studies	[54, 55, 57–60]
Gamma-aminobutyric acid	GABA	Neurons, astrocytes	Most widely distributed inhibitory amino acid neurotransmitter; dysregulation results in motor, memory and cognitive disorders; association between GABA concentration changes and progressive stroke or neurological deterioration especially in lacunar infarcts	[61–69]
Serine	Ser	Neurons, astrocytes	Physiological function of Ser relays on co-activation of the NMDA receptors; based on its transient increase after reversible cerebral ischemia, its value as an indicator of threatening glutamatergic excitotoxicity can be considered	[70, 71]
Glutamine synthetase	GS	Astrocytes	Enzyme responsible for maintaining of glutamate-glutamine balance, reveals increased expression after transient cerebral ischemia; its diagnostic value in assessment of threatening glutamatergic excitotoxicity and size of brain tissue damage can be considered	[72–75]
Serine racemase	SR	Neurons, astrocytes	Enzyme isomerizing l-serine into d-serine; diagnostic significance as an indirect marker of glutamatergic excitotoxicity and brain tissue damage can be considered	[71, 76]
Neuron-specific enolase	NSE	Neurons	Glycolytic enzyme, changing expression in acute phase of ischemic stroke; correlation reported between the NSE serum level and ischemic infarct volume; indicator of ischemic infarct hemorrhagic transformation; predictor of outcome after complications resulting from diagnostic and therapeutic procedures	[77–82]
Inflammatory mediators				
Interleukin-6	Il-6	Astrocytes, microglia	Proinflammatory cytokine regulating release of inflammatory mediators, apoptosis and BBB integrity; association of its concentration with increase of ischemic infarct volume; predictor of risk estimation and outcome prognosis in progressive stroke, TIA and lacunar stroke	[82–88]
Matrix metalloproteinase-9	MMP-9	Neurons, astrocytes, microglia, macrophages, pericytes and endothelial cells	Collagenase responsible for breakdown of extracellular matrix; its increased level in acute ischemic stroke is associated with higher risk of neurological deficits, infarct hemorrhagic transformation and mortality; positive role of MMP-9 in stroke recovery is discussed	[89–96]
Tumor necrosis factor α	TNFα	Macrophages, neurons	Factor enhancing astrocytic and microglial inflammatory response in ischemia-reperfusion injury, also involved in regulation of cell death and glutamatergic excitotoxicity; valuable in assessment of stroke with accompanying metabolic disorders; diagnostic value for prediction of neurological status after hypoxia-ischemia injury in newborns and carotid artery stenting	[97–104]
von Willebrand factor	vWF	Endothelial cells, megakaryocytes	Glycoprotein involved in platelet adhesion and aggregation, influences inflammatory response through regulation of cytokine release; biomarker of diagnostic value in acute phase of all types of ischemic stroke, also related with increased risk of the first ischemic stroke, poor neurological outcome and hemorrhagic transformation of ischemic infarct	[25, 105–108]
Interleukin-4	Il-4	Astrocytes, microglia	Cytokine of anti-inflammatory function, contributing to reduction of acute ischemic damage and infarct volume; regulates expression of anti-inflammatory mediators; considered potential predictor of neurological status in acute phase of ischemic stroke	[109–111]
Interleukin-10	Il-10	Astrocytes, microglia	Anti-inflammatory and neuroprotective cytokine, inhibits neuronal apoptosis; considered predictor of neurological status of patients with small-vessel disease	[88, 112–114]
Neurotrophic and growth factors				
Brain-derived neurotrophic factor	BDNF	Neurons, astrocytes, microglia, ependymal and endothelial cells	Modulator of signaling pathways and cytokines release; prognostic significance for evaluation of functional outcome after ischemic stroke and impending post-stroke depression or cognitive impairment is discussed	[115–120]
Glial cell line–derived neurotrophic factor	GDNF	Astrocytes	Modulator of inflammatory response and astrogliosis, improving post-stroke recovery; alleviates learning and memory disorders; can be considered a potential biomarker of neuroprotective processes and BBB status	[121–125]
Nerve growth factor	NGF	Astrocytes, pericytes	Inhibitor of apoptosis, enhancing cognitive and memory processes after transient cerebral ischemia in vivo; assessment of clinical diagnostic value is inconclusive; further studies are necessary to determine significance of this biomarker in different types of stroke	[126–129]

## Neuroglial and Neuronal Structural Proteins

### Glial Fibrillary Acidic Protein

GFAP is a valuable marker of cellular integrity. Changes in expression of this astrocytic cytoskeleton protein are strongly dependent on the intensity of reactive gliosis [29, 35]. Upregulation of GFAP has been found not only in the course of ischemic but also in the hemorrhagic stroke. In the latter, the increase in expression is even higher than that in the ischemic infarct [36, 37]. In the course of the hemorrhagic stroke, its highest serum level occurs between 2 and 6 h [36], whereas after ischemic stroke onset the increase in serum concentration raises after 24 h and reaches maximal level between the second and the third day [131]. However, correlation between the size of lesion and GFAP serum concentration does exist in the case of hemorrhagic stroke and not for the ischemic stroke, but only after 2 h from its onset. This can be explained by initially small volume of lesioned tissue, which increases with the time lapse [132]. Therefore, in the time window between 2 and 6 h after stroke onset, GFAP could be considered useful for differentiation between hemorrhagic and ischemic stroke. However, during the first 2 h, GFAP sensitivity for detecting hemorrhagic stroke is low, which makes this marker useless in diagnosis of the earliest phase of this pathology. Interestingly, the difference in GFAP expression between hemorrhagic and ischemic stroke has not been confirmed in Chinese cohort study, which provides evidence for existing ethnic differences that should be taken into consideration by analyzing different population studies [133]. There is also evidence indicating a correlation between GFAP concentration in CSF and the National Institutes of Health Stroke Scale (NIHSS) score in patients during acute phase of stroke, and a correlation between this marker level and the infarct volume, as well as modified Rankin Scale (mRS) score after three months from the stroke onset [38].

In addition to being useful in diagnosing hemorrhagic stroke, GFAP could have some value in diagnosing early phase of traumatic brain injury [39]. Due to its high sensitivity (56–89%) and specificity (100%), it is a useful indicator of traumatic brain injury, helpful in assessing the patient’s early condition after injury or choosing further diagnostic methods and therapy. However, it should be taken into account that its concentration decreases after 6 h [132].

Overall, GFAP cannot be considered a specific marker of hemorrhagic or ischemic stroke during the very early phase (0–2 h). The difference in the dynamics of its expression in the early stages (between 2 and 6 h) of the course of both forms of stroke can, however, shed light on the etiology (hemorrhagic or ischemic) and thus, in combination with the data from the patient’s history and the results of the neurological examination and radiological tests (if available), can facilitate differentiating diagnosis. Despite the increasing availability of neuroimaging tests, there is still a fairly large group of patients, in whom these examinations were not performed in the early stages of stroke or clinical evaluation in pre-hospital settings was undertaken by a non-specialist. In such cases, an assessment of GFAP level and dynamics of its changes may be useful in diagnostic process and considering most appropriate therapy. The characteristic difference in dynamics of its concentration changes may provide some help in differentiating diagnosis in the absence of precise data on the time point of stroke onset. In addition, there are premises indicating GFAP value in determining outcome prognosis and neurological status of stroke patients. In summary, GFAP cannot be considered a characteristic marker of only one type of stroke and is also activated in the course of traumatic brain injury. However, different dynamics of its concentration changes in the blood serum, association with the size of tissue damage, and patients’ outcome indicate the diagnostic value. Determining possibilities of its clinical application requires further studies.

### S100 Beta Protein

The S100β protein, a representative of the S100-calmodulin-troponin cytoplasmic proteins family, is present in astrocytes, oligodendrocytes, and Schwann cells. The S100β protein reveals neuroprotective or neurotoxic function, depending on its concentration level [40, 41]. In the nanomolar concentrations, it supports neurogenesis and neuronal differentiation [134]. This role of S100β protein is associated with the regenerative processes occurring after stroke (e.g., in hippocampus), as well as with postischemic neuroplasticity. These processes can be responsible for improvement of cognitive and memory functions after ischemic stroke [40, 134]. In higher concentrations, the S100β protein stimulates the release of nitric oxide (NO) and NF-κB and promotes both necrotic and apoptotic cell death [42, 135, 136]. Changes of the S100β protein expression are to the lesser degree dependent on the intensity of reactive gliosis, compared to GFAP. The correlation between the S100β protein serum level and the infarct volume has been revealed between the first and the fourth day of ischemia [137–139]. The S100β protein leakage from astrocytes starts after 4 h from the ischemia onset and depends from depth of the cerebral blood flow reduction and tissue destruction. An early increase of the S100β protein serum levels has been reported to be associated with the malignant form of ischemic stroke, characterized by occurrence of clinical signs of cerebral herniation, loss of brain stem reflexes, and death within 7 days after symptom onset after proximal MCA occlusion [43]. A single measurement of S100β protein in serum between 12 and 24 h after ischemia onset has a predictive value and can be a valuable confirmation of the results assessed on the basis of neurological examination and neuroradiological studies. In the course of cerebral ischemic stroke and TIA, an increase in concentration of S100β protein in CSF was detected within about 9 h from the stroke symptoms onset [38]. Aurell et al. observed an increase in S100β in CSF within 48 h from stroke symptoms onset. This increase was transiently sustained for one week and decreased subsequently [44]. Additionally, in this study, a significant correlation was found between the CSF S100β protein concentration and the infarct size. The CSF S100β levels positively correlate with stroke severity, assessed with the NIHSS score on admission, also with an infarct volume, and with patients’ outcome according to the mRS score after three months from the ischemic infarct onset [38]. S100β protein levels in CSF are differentiated in various types of stroke, being higher in cardioembolic stroke, compared to the small-vessel disease, and they correlate with an infarct volume [45].

In summary, the unique significance of S100β protein as a potential biomarker of ischemic stroke results from the fact that it undergoes early upregulation in the course of brain tissue damage (as in the case of malignant stroke) and is a good predictor of dynamically occurring destructive processes. Depending on the extent of cerebral blood flow reduction, gradual transition of penumbra into the ischemic core can occur very dynamically, within the first 24 h from ischemia onset. In a significant proportion of cases, the ischemic “at risk zone” is not visible on CT imaging, even up to 24–48 h from its onset [139]. In these cases, an increase in concentration of S100β protein may serve as an early warning signal before it becomes visible in the CT image (if available) in the early phase of stroke. Hence, this biomarker can be helpful for predicting development of malignant stroke, increase of infarct volume, or exclusion of stroke mimics.

### Aquaporin-4

AQP4 is a member of the water-channel proteins family, expressed in the end-foot of astrocytes, which surround capillary vessels, ventricular walls or subarachnoid space [140]. AQP4 reveals different effects upon brain tissue, depending on the prevailing type of brain edema. It facilitates astrocytic swelling in the course of cytotoxic edema and enhances extracellular water clearance in vasogenic edema [46, 47]. AQP4 expression pattern in the course of brain ischemia may be different [141]. It can be either up- or downregulated, as a result of the extent of astrocytic damage, reaching critical threshold level. In both cases, changes in AQP4 level are associated with blood vessel wall damage, BBB disintegration, and its increased permeability. There is evidence indicating that AQP4 deficit results in tight junctions opening and perivascular astrocytic end-feet swallowing, which promote development of brain edema [142]. AQP4 also contributes to regulation of regional cerebral blood flow in capillaries [143]. Inhibition of AQP4 expression significantly increases regional cerebral blood flow [144]. All these studies prove AQP4 involvement in development of the malignant brain edema or hemorrhagic transformation of ischemic infarct and justify its value as a potential biomarker. The increase of the AQP4 expression after transient ischemic stroke has been observed between the first and third day of reperfusion [145]. However, the direct assessment of the AQP4 content in the blood serum has not been proved as useful for stroke diagnosis. Importantly, the changes in AQP4 expression and concentration level during brain edema correlate with the MRI results of diffusion-weighted imaging (DWI) and T2-weighted imaging (T2WI) studies [48].

Badaut et al. [28] showed in a rat pup stroke model that upregulation of AQP4 protein expression on astrocytic end-feet (studied with immunohistochemistry) correlated with edema formation assessed with the high-field MRI. This observation should be taken into account when considering diagnostic application of AQP4 in clinical practice. In particular, this correlation can provide some valuable data concerning evolution of the ischemic area and development of potential complications, such as malignant stroke or hemorrhagic transformation of the lesioned region. Similarly, the PET imaging studies have been claimed to reveal an association with distribution and concentration changes of AQP4 in different brain areas, in particular, in the subpial or perivascular regions and in the choroid plexus [49]. Although at present, PET is not a standard method used to assess the content of AQP4 in the CNS. However, it could provide valuable information based on AQP4 function in the course of ischemic process and its uneven distribution in the brain. This could be of importance when considering AQP4 as diagnostic marker or potential therapeutic target [49].

The abovementioned data are of important practical value for elaboration of future diagnostic protocols, based on the distribution of ischemic biomarkers and results of functional neuroradiological imaging studies. This approach, based on evaluation of biomarkers’ concentration and distribution, together with the results of neuroradiological studies, could also enable assessment of status of physiological brain barriers (e.g., BBB, blood/CSF barrier, or brain/CSF barrier) in the course of ischemic stroke.

### Fatty Acid–Binding proteins

Fatty acid–binding proteins (FABPs) represent a family of cytoplasmic proteins, responsible for maintaining an appropriate serum concentration of the long-chain fatty acids and expression of genes regulating cellular growth and differentiation [50, 146]. Two of nine proteins from this family, namely H-FABP and B-FABP (detected in neurons and neuroglia, respectively), are of some importance for the cerebral stroke diagnosis [51, 52]. Studies have shown a correlation between a H-FABP blood serum concentration and size of the ischemic infarct evaluated by CT studies, especially when the infarct volume had exceeded 150 ml [50]. Moreover, an association has been found between H-FABP serum levels and severity of neurological deficits, as well as functional outcome assessed with the NIHSS and mRS. The highest serum concentrations of another fatty acid–binding protein, B-FABP, were found in lacunar and small subcortical infarcts of 1–50 ml volume. In contrast to H-FABP, no correlation was found between B-FABP serum levels and neurological deficits in stroke patients. Lack of correlation between NIHSS or mRS and B-FABP level may result from minor damage to the nervous tissue. Moreover, distribution of B-FABP predominantly in neuroglial cells (in contrast to H-FABP found mainly in neurons) can explain the different nature of brain tissue lesion, associated to a greater extent with neuroglial than neuronal damage. Consequently, this may be responsible for less pronounced neurological deficits and the lack of correlation between the B-FABP level and patient’s clinical condition, assessed by NIHSS and mRS scores.

The advantage of using both FABPs as potential stroke biomarkers is related with early increase of their serum concentrations during stroke, which occurs already within 2 to 3 h after ischemia and remains elevated up to 120 h. The disadvantage is concerned with their relatively low specificity, whereas increase in H-FABP concentration has also been observed in the hemorrhagic stroke [53]. The serum levels of both these markers can be indicators of stroke damage size. Indeed, simultaneous assessment of both biomarkers provides greater benefit allowing to draw conclusions not only as to size of the damaged area, correlation with functional deficit, and prognosis (high level of H-FABP), but also as to the type of stroke (e.g., lacunar or small-vessel disease, when high level of B-FABP). In the early phase of both ischemic and hemorrhagic stroke, small size of damage may be the reason why changes in both biomarkers’ concentration are not specific enough to allow for clear differentiation between these two forms. While low level of H-FABP and high level of B-FABP do not allow for differentiation between hemorrhagic and ischemic stroke, some other information could be obtained based on changes in these biomarkers such as the time point of stroke onset (upregulation even within 2–3 h after stroke onset), nature of tissue damage (rather neuroglial than neuronal lesion), size of lesion, and pathophysiological type of stroke (more likely lacunar than large ischemic infarct). In such a case, diagnosis may be supported by assessment of the other markers used in the diagnostic panel, accompanied by neurological status assessment, and results of neuroradiological examinations.

Reassuming, perspectives of diagnostic application of FABPs as ischemic stroke biomarkers are promising, although require further investigation. Their advantage is in principle concerned with rapid increase of serum concentration and multifunctional value, enabling indirect assessment of the volume of ischemic lesion, severity of neurological deficits, and differentiation among types of ischemic stroke.

## Amino Acid Neurotransmitters and Enzymes

The results of numerous studies indicate changes in concentration of both excitatory and inhibitory amino acid neurotransmitters, such as glutamate (Glu) and serine (Ser) or γ-aminobutyric acid (GABA) and glycine (Gly), respectively, in the course of ischemic stroke. These changes may be a good indicator of the depth of neuronal and neuroglial dysfunction. They may also correlate with the severity of neurological deficits, of both focal character (e.g., hemiparesis, aphasia, or visual deficits) and general nature (e.g., consciousness disturbances or memory and cognitive disorders), depending on localization of the lesioned area. However, dynamic changes in concentration of neurotransmitters, in the course of various types of ischemic stroke, are poorly understood. Further studies on these processes will provide important information concerning dysfunction of particular brain structures affected by ischemia. Another group of potential biomarkers, deriving from the NVU, is represented by enzymes responsible for synthetizing of neurotransmitters and metabolites significant for diagnosis of the ischemic stroke. Among these, one could encounter glutamine synthetase (GS), serine racemase (SR), and neuron-specific enolase (NSE). Their potential role as stroke biomarkers is presented in the following paragraphs.

### Glutamate

In ischemic stroke, an increase in Glu concentration in both blood plasma and CSF was observed. Due to the easy passage through the cerebral functional barriers, these changes were positively correlated, which implies diagnostic value of Glu concentration measurement in both physiological fluids [54, 55]. A progression of neurological deficits and early neurological deterioration, within 72 h in stroke patients, was observed accompanied by increased concentrations of Glu in both of them.

There is well-documented evidence showing characteristic difference in dynamics of Glu level changes between patients with stable and progressive ischemic stroke [54, 56]. While in the former there is only a slight Glu increase terminating within 6 h, in the latter, Glu concentration increases considerably and remains elevated for at least 24 h. This has been also observed in patients with a progressive brain swelling diagnosed in CT examination. Glu concentrations exceeding 200 μmol/l in blood plasma and 8.2 μmol/l in CSF are associated with progression of neurological deficit within 48 h. Diagnostic value of Glu concentration assessment in blood plasma and in CSF may also result from correlation between its content and infarct volume. This can indicate the increased risk of poor prognosis in patients with high Glu levels. Reassuming, further studies on diagnostic application of Glu level assessment seem very promising. They should be continued concentrating among others on evaluation of Glu concentration changes in different types of ischemic stroke, as well as on relationship between Glu levels and dynamics of neurological deficit changes.

### Glycine

It has been shown that Gly concentration in blood plasma and CSF increases in stroke patients, correlating with progression of neurological deficits [54, 55]. As a neurotransmitter present at relatively high concentration in numerous CNS structures, Gly is involved in regulation of neuronal activity in forebrain, brain stem, and spinal cord [57]. The inhibitory action of Gly observed in physiological conditions in different brain areas can be disturbed by ischemia. Increase or decrease of Gly concentration can result in appearance of neurological symptoms in the form of functional deficits or excitation, respectively. In the first case, it can augment motor or sensory deficits [58, 147]; in the second, it can promote conditions initiating epileptic seizures or muscle tone disorders [59]. Some evidence indicates that plasma and CSF concentrations of Gly are higher in patients with progressive ischemic stroke, compared to those with its stabile variant, as well as, that their increased concentrations (plasma Gly > 223 μmol/l; CSF Gly > 126 μmol/l) are present in patients with progression of neurological deficits [54, 55]. Results of a recent study show that Gly level assessment can be useful for differentiating patients with ischemic stroke from the control group [60]. Reassuming, all these data suggest that Gly could be a useful biomarker, included in new stroke diagnostic panels, aimed at differentiating ischemic stroke from negative control and differentiating its stabile variant from progressive one.

### γ-Aminobutyric Acid

Concentration of this inhibitory neurotransmitter also reveals characteristic changes in the course of different types of ischemic stroke. Considering the widespread distribution of GABA in cerebral structures, in particular, in areas related to motor function, such as frontal cortex or basal ganglia (e.g., striatum, globus pallidus, subthalamic nucleus and substantia nigra), it can be expected that dysregulation of GABA-ergic neurotransmission, resulting from ischemia, may be responsible for enhancing of disorders, such as paresis, muscular tone dysregulation, or involuntary movements [61]. It can be also responsible for initiation of the epileptic activity [62]. Other brain areas in which GABA-ergic transmission disorders can bring about characteristic dysfunctions are limbic structures, such as hippocampus, amygdala, or prefrontal cortex. Disturbance of the balance between inhibitory and excitatory neurotransmission in these areas can lead to increasing cognitive and memory deficits, pathological emotional disorders (e.g., anxiety), or improper behavioral reactions [63–65]. The relationship has been demonstrated between GABA content and neurological deterioration in the early phase of ischemic stroke [66]. The progressive character of ischemic stroke causes a greater decrease of GABA concentration than in the case of a stable stroke. This can have a predictive value for evaluation of neurological status. Changes in the GABA content have been observed within 24 h after transient ischemia onset [67, 68]. In ischemic stroke, the initial increase in GABA concentration is followed by subsequent inhibition of GABA synthesis, leading to GABA-ergic neurotransmission dysfunction, persisting even for the next 3 months [148]. This could explain the observations of some other authors that serum concentration of GABA has not been proved useful as prognostic factor for prediction of ischemic stroke outcome when assessed early after symptoms’ onset [149]. The reduction of tonic GABA-ergic inhibition, especially in the early postischemic period, apart from glutamatergic excitotoxicity, may enhance disturbances of synaptic plasticity. However, subsequently occurring increase in GABA content, through limiting damage, can be encountered among the neuroprotective processes [150]. Furthermore, increased tonic GABA-related inhibition, mediated presumably by GABA A receptors, might lead to improved motor function after ischemic stroke [151]. The correlation between concentration of GABA in blood plasma and CSF is poor [152]. This can be explained by the diversity of mechanisms associated with GABA transition through the physiological barriers in the brain after the onset of ischemia. The reduction in the inhibitory effect of GABA during ischemia is not only a consequence of changes in concentration of neurotransmitter itself, but also result of the response induced by GABAergic receptors [153]. During ischemia, the expression of GABA A and GABA B receptors is reduced and their endocytosis is initiated [154]. This weakens the inhibitory GABA-ergic response, thus enhancing the effect of glutamatergic excitotoxicity [153, 155]. On the other hand, if during ischemia the GABA concentration is retained on the higher level in certain brain areas, simultaneous stimulation of GABA A and GABA B receptors results in neuroprotective effect [156]. Regulation of GABA-ergic receptors’ trafficking, their surface expression and recycling, modulate the strength of GABA-ergic synaptic inhibition [157]. GABA-ergic neurotransmission disorders affect the balance between excitation and inhibition of neurons in the area of damage [158]. The large body of evidence indicates reciprocally related changes in GABA and Glu content in the course of ischemia. This could be due to the interrelated biochemical transformation pathways of both these amino acid neurotransmitters, approximately the same time period of their release into the ischemic area, and their involvement in regulation of pathophysiological processes occurring in the early phase of ischemia. Changes in concentration of each of them affect the extent of damage to the ischemic tissue [69]. The blood plasma Glu concentration > 200 μmol/l and GABA concentration < 240 nmol/l can be considered predictors of early neurological deterioration in the course of lacunar infarction [66]. A decrease in GABA content in the plasma, with concomitant increase in Glu level, results in increased Glu/GABA ratio, which is characteristic for glutamatergic excitotoxicity. Moreover, a Glu/GABA ratio higher than 106 is regarded as a predictor of early neurological deterioration in 85% of patients with lacunar infarction. Subsequent increase in GABA secretion leading to reduction of this ratio signals lowering of the intensity of damage and promotion of reparative processes [150]. The Glu/GABA ratio can differ between the infarct core and peripherally localized penumbra [159]. Thus, the assessment of Glu/GABA ratio, along with the evolution of changes in the ischemic area observed in neuroradiological studies, may become useful diagnostic and prognostic tool.

In summary, decrease in GABA concentration in the blood plasma could be a predictor of the worse outcome in patients with progressive stroke. Demonstration of the relationship between GABA and Glu concentration changes, localization of the ischemic area, and severity of functional disorders in the course of ischemia can provide interesting data supplementing diagnosis and prediction of complications in the course of ischemic stroke.

### Serine

This amino acid co-neurotransmitter and gliotransmitter is widely distributed in the CNS in two isoforms, of which d-Ser is converted from l-Ser in neurons and astrocytes [160, 161]. Physiological function of d-Ser relays on binding to Gly site and co-activating NMDA receptors, whereas overstimulation can result in glutamatergic excitotoxicity. After transient ischemia, d- and l-Ser blood concentrations transiently increase, reaching their maximal levels at 20 h, which suggests their role as biomarkers of the acute phase of ischemia [70]. Some authors point to potentially neuroprotective effect resulting from reduced d-Ser binding to the NMDA receptor, which has been described in pathological processes related to reduced synthesis of Ser [71]. This results in decreased NO and Glu release and in consequence, in reduced infarct volume. Reassuming, although the role of Ser in various types of ischemic stroke and its different phases has not been extensively studied, dynamic changes in its content, especially in the early phase of ischemia, and the widespread regulation based on NMDA receptors, indicate the importance of assessment of Ser concentration changes. Monitoring of this amino acid levels could have some prognostic value as an indirect indicator of threatening glutamatergic excitotoxicity or neuroprotective processes, depending on its concentration levels.

### Glutamine Synthetase

This glutamine (Gln) synthetizing astrocytic enzyme is responsible for maintaining of Glu/Gln balance in physiological conditions [162]. It is sensitive to free oxygen and nitrogen radicals that inactivate it when released during the early phase of ischemia [72, 73]. However, GS activity is rapidly restored, especially after transient ischemia [73, 74]. The GS immunoreactivity has been reported to be increased within 3 h of postischemic reperfusion [75]. Due to its involvement in dysregulation of Glu/Gln balance and transient increase in Glu levels, GS is indirectly responsible for destructive effects of Glu excitotoxicity. Hence, GS activity level and concentration assessment during ischemia can predict the deleterious effects of Glu exerted upon infarcted area. This indicates direction of further research on this enzyme, as an indicator of an early neurological deterioration in the course of transient ischemia. Overall, dynamic character of GS activity, its expression changes and involvement in regulation of the Glu/Gln balance, suggest an important role of this enzyme in ischemic stroke and justify further studies.

### Serine Racemase

Function of SR is concerned with isomerizing of l-Ser into d-Ser [163]. Recent studies have shown that mRNA and protein levels of SR are higher in neurons than in astrocytes [164]. In mouse model of permanent ischemia, the SR mRNA and protein expression decrease between the 6th and 10th day after stroke [76]. The exact explanation of downregulation of SR in subacute phase of ischemia is unknown. It has been speculated that it could be a consequence of transient decrease in its transcription. This is consistent with the D-Ser role in stimulation of NMDA receptors, presumably in the acute phase of brain ischemia, resulting in maximal excitotoxic damage. Hence, the decrease in SR protein and mRNA levels during subacute phase could be related with cellular death, resulting from glutamatergic excitotoxicity. Changes of the SR expression could be taken into account as indirect indicator of glutamatergic excitotoxicity and destructive processes [71]. However, no clinical results confirming the SR diagnostic value are available and additional studies are required to verify its significance as a potential biomarker.

### Neuron-Specific Enolase

NSE is an isoform of glycolytic enzyme, which expression changes in acute phase of ischemic stroke [77]. In this process, the NSE serum levels are higher, compared to the hemorrhagic stroke and positive correlation has been reported between the ischemic infarct area and the NSE serum levels in acute phase of stroke. In contrast, no association has been found between NSE levels and hemorrhagic infarct volume [165]. Dynamic changes of the NSE serum levels in acute ischemic stroke are predictive for its hemorrhagic transformation and BBB disruption [78]. An association has also been found between the NSE serum levels and severity of cerebral venous thrombosis and NSE is regarded as a predictor of clinical outcome in this process [166]. Evaluation of the NSE levels has been recommended as a valuable indicator of poor outcome or death in the course of hypoxic-ischemic brain lesion after cardiac arrest [79, 167]. Complications occurring after diagnostic and therapeutic procedures are also concerned with changes in the NSE levels. The increase of its serum levels at 48 h has a predictive value in the course of ischemia-reperfusion brain damage after internal carotid endarterectomy [80]. This enzyme could also be useful for diagnosis of the silent cerebral infarcts caused by microemboli in the course of invasive coronary procedures [81]. Finally, an association has been found between the lower NSE concentration and favorable outcome after intravenous thrombolysis [82]. Reassuming, the role of NSE in the course of cerebral stroke is complex. Changes of its concentration are associated with destructive processes affecting brain tissue during cerebral stroke, such as BBB disruption and hemorrhagic transformation. Its involvement as potential indicator of complications, which occur during diagnostic and therapeutic procedures, justify the need for further studies on this enzyme.

## Inflammatory Mediators

Another group of potential ischemic stroke markers consists of pro- and anti-inflammatory mediators. Majority of these biomarkers is not characteristic for one cellular subpopulation but rather for all constituents of NVU [168, 169]. The proinflammatory and neurodegenerative mediators are represented by cytokines (interleukin-6; IL-6), enzymes (matrix metalloproteinase-9; MMP-9), neurodegenerative factors (tumor necrosis factor α; TNFα), and hemostatic factors (von Willebrand factor; vWF). Among the anti-inflammatory cytokines could be encountered interleukin-4 (IL-4) and interleukin-10 (IL-10).

### Interleukin-6

IL-6 is a proinflammatory cytokine secreted by microglia or astrocytes, depending on the acute or subacute phase of ischemic stroke, respectively [170, 171]. This interleukin reveals modulatory effect upon apoptosis, the BBB integrity, and secretion of other inflammatory cytokines [82]. Its serum levels increase in the course of ischemic stroke, and together with the infarct size assessment, this factor can be used as a valuable predictor of stroke prognosis [83, 84]. Elevated concentration of IL-6 at 6 h and increased NIHSS score on admission were associated with early deterioration within 48 h or death. At 72 h from the ischemic infarct onset, IL-6 concentration correlated with the S100β protein levels and size of tissue damage [172]. The IL-6 serum levels changes could also be useful for evaluation of transient ischemic attack (TIA) and silent lacunar infarction. Studies revealed prognostic value of IL-6 for the risk estimation in TIA and first-ever cerebrovascular events [85, 86]. Some recent studies also have shown higher levels of IL-6 in the course of large-artery stroke, compared to the lacunar stroke [172]. Changes in IL-6 serum concentration are not characteristic exclusively for ischemic infarcts and have been also found in the course of subarachnoid hemorrhage and hemorrhagic stroke [173, 174]. Higher levels of IL-6 detected between 3 and 7 days after subarachnoid hemorrhage were associated with delayed ischemic neurological deficits and unfavorable outcome. In hemorrhagic stroke, IL-6 level was increased between days 1 and 4, although the level was lower compared to the ischemic stroke [175]. High levels of IL-6 in the acute phase of hemorrhagic stroke correlated with the severity of the cerebral edema and indicated a poor neurological outcome [87, 88]. The results of the abovementioned studies indicate that role of IL-6 in the pathomechanism of inflammatory response in various forms of cerebral infarction could be more complex than previously assumed and confirm the need for further studies, both in the experimental models and clinical settings. The relationship between IL-6 gene polymorphism and increased risk of cerebral infarction has been suggested [176, 177], although this was not confirmed by the other published results and further studies of this issue are required [178].

### Matrix Metalloproteinase-9

MMP-9 is an inducible enzyme, representing a large family of over 20 proteinases, involved in degradation of the extracellular matrix and secreted by different components of NVU. In the course of acute ischemic stroke, MMP-9 function is related to disintegration of the components of extracellular matrix and disruption of BBB, through degradation of the basal lamina, in particular laminin, type IV collagen, and fibronectin [89, 179]. Compromising of BBB integrity results in extravasation of blood plasma and cells and in development of vasogenic edema and hemorrhagic transformation. This is especially the case when augmented by reperfusion in the ischemic tissue, resulting from dissolution of the embolus [180]. The results of experimental studies and clinical observations indicate the high level of MMP-9 to be associated not only with the infarct volume increase and progress of neurologic deficits but also with hemorrhagic transformation [89]. In the mouse model of transient ischemia, an increase of the MMP-9 expression was observed after 4 h of reperfusion and coexisted with the increase of infarct volume and neurological deficits [90]. Clinical study conducted on the group of 3186 patients with acute ischemic stroke revealed elevated MMP-9 serum levels, which were associated with higher risk of mortality and major disability [91]. Recently, it has been reported that MMP-9 can be used as a marker of the hemorrhagic transformation in the course of acute ischemic stroke [89]. On the basis of meta-analysis published by Wang and colleagues, the pooled sensitivity and specificity values of MMP-9 in predicting of hemorrhagic transformation in the course of acute ischemic stroke are 85% and 79%, respectively [89]. Castellanos et al. [92] showed that plasma MMP-9 concentration on admission in patients with ischemic stroke who developed hemorrhagic transformation was significantly higher than in those who did not develop this complication, as well as in those from the control group. A predictive value of increased level of MMP-9, regarding threatening intracerebral hemorrhage, was raised in patients treated with thrombolytic therapy [93, 179]. Significantly increased plasma MMP-9 concentration (191.4 ng/ml) was detected in patients who later suffered from the parenchymal intracerebral hemorrhage in comparison to the control group (68.05 ng/ml) [93, 94]. Validation of the predictive capacity of MMP-9 pre-established cutoff value (≥ 140 ng/ml) for parenchymal hemorrhage development after thrombolytic therapy has been proved with sensitivity of 92% and specificity of 74% [95]. Characteristic MMP-9 concentration changes have been also revealed in the course of cardioembolic stroke [180]. The increased baseline values for MMP-9 levels can be treated as a predictor of hemorrhagic transformation which occurs between the 5th and 7th day, whereas peak of MMP-9 at 24 h time point can be a predictor for the early (occurring within 48 h) parenchymal hematoma development. Apart from these, there is also some evidence suggesting positive role of MMP-9 in stroke recovery. Recent study by Abdelnaseer and colleagues [96] has shown increased MMP-9 levels during 30 days after stroke onset, which correlated with the improved patients’ clinical status. However, explanation of the MMP-9 role in this phenomenon requires further studies. The MMP-9 could be also a useful biomarker for identification of threatening vasospasm following subarachnoid hemorrhage [181] and a predictor of delayed cerebral ischemia [182]. Some evidence indicates the MMP-9 gene polymorphism associated with the increased risk of ischemic stroke [183, 184]. Altogether, changes of the MMP-9 serum levels in the course of ischemic stroke are a signal of several pathophysiological processes, which determines its diagnostic value. Considering the well-known negative effects of MMP-9 in the course of stroke, as well as some evidence indicating its positive function, it can be assumed that place of this marker in diagnosis of stroke is still not precisely defined and requires further basic and clinical studies.

### Tumor Necrosis Factor α

TNFα is expressed by macrophages and neurons in the infarcted area that enhances astrocytic and microglial inflammatory response in the course of ischemia-reperfusion injury [97]. Its function is related to regulation of the cell death and inflammatory response [98, 185] and enhancement of glutamatergic excitotoxicity [99]. Through the NFκB-dependent signaling pathway, this cytokine can influence proliferative potential of the reactive astrocytes [186, 187]. An elevated level of the TNFα in blood serum has been revealed as a predictor of deteriorating neurological status of newborns after hypoxia-ischemia injury [100]. Other study has shown that TNFα could be a good indicator of the increased risk of ischemic brain damage after carotid artery stenting [101]. There is also well-documented evidence indicating that monitoring of TNFα and IL-1β levels can provide some valuable information allowing for diagnosis of stroke in patients with accompanying metabolic disorders and those without such complications. In patients with acute ischemic stroke and metabolic syndrome, higher TNFα and IL-1β activation were observed, independently of stroke type (such as large artery atherosclerosis, lacunar and cardioembolic infarct), when compared to patients with the same types of stroke but without metabolic syndrome [102]. In the latter, the highest level of inflammatory response was observed in the cardioembolic stroke and the lowest in lacunar stroke. Additionally, an association has been found between the severity of neurological deficits on admission, intensity of inflammatory response, and stroke type [103]. In diabetic patients with lacunar stroke, activation levels of TNFα and IL-1β in the acute phase (24–72 h) and subacute phase (7–10 days) of stroke were lower, compared to non-diabetic patients, which could be associated with their better outcome [104]. Hence, TNFα and IL-1β plasma level assessment can be useful not only for diagnosis of different stroke types, but also their severity and accompanying metabolic disorders. Although some evidence suggests that TNFα gene polymorphism could be a predictor of the recurrent TIAs [188], the value of this factor for prediction of stroke risk is unequivocally assessed and requires further studies [189].

### von Willebrand factor

vWF is a hemostatic agent secreted by the endothelial cells and megakaryocytes. It is involved in platelet adhesion, aggregation, and activation of complement system [190]. The vWF is a mediator of vascular inflammation which affects the release of IL-1β, and TNFα [25]. The role of this factor as an ischemic biomarker has been extensively studied [191]. The elevated vWF serum levels have been reported as a predictor of an increased risk of the first ischemic stroke [105]. Other studies showed that vWF serum levels were higher in patients with the acute stroke and TIA than in those with chronic cerebrovascular disease [106, 192]. According to the Trial of Org 10172 in Acute Stroke Treatment (TOAST) classification, levels of vWF increase in all types of stroke in its acute phase, although after 3 months its increased concentration remains in the cardioembolic and cryptogenic stroke [107]. An association between elevated levels of vWF and the NIHSS score on admission and poor clinical outcome has been demonstrated, although not for the cardioembolic stroke [108]. The increase of the vWF levels has also been reported in the course of symptomatic carotid stenosis [106]. Reassuming, a coincidence between increased level of vWF and different types of ischemic stroke has been confirmed, making this factor a potential risk indicator of cerebrovascular diseases. What seems practically important is that the vWF levels differ among various clinical and etiological subtypes of ischemic stroke, which must be taken into account when conducting laboratory and clinical studies on this promising biomarker. High levels of vWF are associated with severity of stroke, as well as with poor clinical outcome.

### Interleukin-4

This anti-inflammatory cytokine contributes to reduction of the acute ischemic damage and infarct volume, through the M2 microglia phenotype stimulation [109, 110]. IL-4 regulates infiltration of macrophages and phagocytic microglia into the ischemic area and an increase of expression of the other anti-inflammatory mediators [193]. Changes of the IL-4 serum concentration, together with increase of the IL-6 serum levels, have been reported as diagnostically valuable in the acute phase of ischemic stroke [194]. An increase of expression of IL-4 receptor (IL-4R) is an independent predictor of worsening of the neurological status at 24 h after infarct onset [111]. Studies on the IL-4 gene polymorphism provide some valuable information concerning prognosis for patients with different types of ischemic stroke and related complications. The IL-4-gene 589C>T polymorphism has a prognostic value for evaluation of developing stroke and prediction of the patient’s functional impairment [195]. The C582T IL-4 gene polymorphism is an independent predictor of the thromboembolic stroke [196].

### Interleukin-10

IL-10 is a cytokine of anti-inflammatory and neuroprotective function, released by astrocytes and microglia. It inhibits the production and secretion of IL-1β [197], TGFβ [198], and TNFα [199]. Other functions of this cytokine are related with suppression of neuronal apoptosis [200], neuroglial differentiation, and proliferation [201]. The IL-10 expression increases in acute phase of ischemic stroke [112]. An association has been revealed between the IL-10 serum levels and clinical status of patients with small-vessel disease and subcortical infarctions [113]. The increased IL-10 serum levels have been revealed in patients with severe neurological impairment assessed by NIHSS score at 48 h after the ischemic stroke onset [114]. However, some recent evidence suggests that IL-10 serum concentration negatively correlates with the increased risk of cerebral infarction [88]. Stroke patients with decreased serum levels of IL-10 (< 925.0 pg/ml) demonstrate neurological deterioration within 72 h from the symptoms onset [112]. Therefore, the significance of IL-10 for assessment of ischemic stroke outcome and risk is ambiguous. These different aspects of IL-10 function in ischemic process are most likely related to its complex etiopathogenesis and depth of ischemic damage [169]. Recent studies have shown that the IL-10 gene polymorphism is associated with the incidence of ischemic stroke [202]. This association is even stronger among the cigarette smokers [203].

## Neurotrophic and Growth Factors

The fourth group of potential stroke biomarkers is represented by the brain-derived neurotrophic factor (BDNF), glial cell line–derived neurotrophic factor (GDNF), and nerve growth factor (NGF). Neurotrophic factors could be considered an interesting supplement among the ischemic stroke diagnostic indicators, which results from their predictive value in assessment of neurological status and threatening complications. However, currently available results concerning dynamics of changes in expression of these markers in various types of stroke are fragmentary and frequently inconclusive.

### Brain-Derived Neurotrophic Factor

BDNF is expressed by several constituents of NVU. Apart from neurons, it is also detected in astrocytes and microglial and ependymal cells, as well as in the endothelial cells of cerebral vessels, which reflects its important regulatory function [204, 205]. In the ischemic stroke, it plays a neuroprotective role, associated mainly with modulation of signaling pathways, cytokine release, and suppression of apoptosis [115]. The results of experimental and clinical studies indicate a relationship between the BDNF serum levels and both functional recovery, as well as ischemic lesion volume [116, 206]. There is evidence indicating that lower BDNF serum level is associated with increased risk of stroke or TIA incidence [116]. Furthermore, a negative correlation was found between serum BDNF concentration on admission and infarct volume, assessed by DWI (*r* = − 0.363; *P* < 0.001) in patients with acute ischemic stroke [206]. Recent studies show that this neurotrophin could be also used as a prognostic factor for prediction of the functional status of patients after ischemic stroke [117, 207]. The functional outcome based on the mRS examination performed on the 90th day after stroke onset was significantly worse in those patients whose BDNF level on the first day after admission was lower than 9.96 ± 5.21 ng/ml [207]. Also, low BDNF levels were associated with poor functional outcome in long-term (2 and 7 years) follow-up studies [117]. The results of animal studies showed the improvement of neurological function after stroke, assessed with the modified Neurological Severity Score, with accompanying increase in BDNF concentration [118]. The BDNF can also be used as a diagnostic marker of the threatening post-stroke complications. Animal studies reveal an association between BDNF expression in hippocampus and intensity of the post-stroke depression [119]. Clinical studies also indicate that BDNF could be an independent prognostic factor of the post-stroke depression. There is evidence indicating that patients suffering from post-stroke depression reveal significantly lower BDNF level than patients not suffering from this complication. Serum BDNF concentration < 5.86 ng/ml within 24 h after stroke onset is predictive for the danger of post-stroke depression development [208]. The results of experimental studies reveal also an association between the severity of cognitive impairment and BDNF concentration [120]. Some authors stress practically important differences in the BDNF levels detected in the whole blood and blood serum, which can result from its considerably high content in the blood cells. This must be taken into account while interpreting the diagnostic results relaying on assessment of the concentration of this neurotrophin [209].

### Glial Cell Line–Derived Neurotrophic Factor

GDNF, being a representative of the transforming growth factor-beta family, is secreted by reactive astrocytes [210]. The results of animal studies suggest a potential value of this factor as a biomarker of neuroprotective and reparative processes. Its neuroprotective function relays among others on suppressing of free radical production. Through attenuation of the brain NO synthase activity, NO concentration is decreased, which prevents from glutamate-induced excitotoxic cell damage and consequently inhibits an expansion of infarcted area. By promoting cell survival, neurite outgrowth, and synaptogenesis, GDNF alleviates the ischemia-induced learning and memory disorders [121]. GDNF modulates astrogliosis and inflammatory response in the ischemic penumbra. One of its important physiological functions is protection against an early and delayed neuronal death [122, 123]. Antiapoptotic effect of GDNF is achieved through reduction of DNA fragmentation, and inhibition of the caspase-dependent pathways. GDNF also induces the expression of anti-apoptotic genes (e.g., bcl-2 and bcl-xL). It enhances an antiautophagic effect within the lesioned brain area due to the antiapoptotic and antiautophagic functions. It modulates the post-stroke recovery and contributes to reduction of deleterious effect of ischemia. Characteristic for GDNF is its dynamic expression pattern [124, 211]. Depending on duration of the cerebral blood flow reduction, transient ischemia induces increase in GDNF mRNA expression lasting from 3 h to 3 days, with the peak of its expression at 6 h. This early increase might be followed by a secondary peak at 72 h. The highest GDNF immunoreactivity in astrocytes is observed between the 3rd and 7th day from the ischemia onset. Appearance of GDNF in the vascular system signalizes massive BBB damage, which is requisite for passage of this large molecule [125]. Overall, relatively early changes in GDNF expression could serve as valuable signal of reparative and neuroregenerative processes occurring within the ischemic area. However, further studies are necessary to verify significance of GDNF as an ischemic stroke biomarker.

### Nerve Growth Factor

NGF is expressed by astrocytes and pericytes after stimulation by inflammatory mediators and proinflammatory cytokines [26, 212]. Neuroprotective function of NGF comprises inhibitory action upon apoptosis, resulting from reduced expression of caspase-3 and upregulation of Bcl-2 expression [126, 213]. It also antagonizes the activity of TNFα and IL-1β [127]. After transient cerebral ischemia in rats, NGF induces synaptogenesis which is related with improvement of cognitive and memory functions [128]. The available results of experimental studies indicate temporary increase of NGF expression detected in the cerebral cortex within 6 h after ischemia onset in mice [126]. In rats, there was temporary decline in the NGF content in brain since 4 h of reperfusion, with a gradual increase to normal levels between the 7th and 14th day. These changes are accompanied by an increase in NGF immunoreactivity in astrocytes [214]. After transient forebrain ischemia in gerbils, the NGF level decreased by 32% at 2 days after ischemia in the CA3 and dentate gyrus, whereas it increased by 50% after 2 weeks. These changes could be explained by cell death in the early phase and astrocytic reactivity in the later phase, respectively [215]. To our knowledge, up until now, there are no results published which refer to changes in NGF concentration in the blood serum of patients after ischemic stroke. However, the results of clinical studies showed decrease in NGF levels in the CSF of patients with ischemic cerebrovascular disease and cognitive impairment which confirms important role of this neurotrophin in neuroprotective processes [129]. Overall, determination of practical value of NGF as an ischemic biomarker requires further research. In particular, assessment of its concentration in the blood serum in various types of ischemic stroke would be required. Considering the involvement of NGF in neuroprotective processes, assessment of its changes could provide important diagnostic data.

## Perspectives for Identification of Effective Biomarkers of the Ischemic Stroke

Although the results of experimental studies on numerous candidate stroke biomarkers and meta-analyses have been presented and discussed in many publications, so far, none of potential biomarkers have met clinical expectations [3, 4, 8, 216, 217]. Among the possible reasons mentioned by the experts in several publications, one can find methodological limitations of some studies, too small groups of analyzed patients, inadequate validation of results, difficulties in determination of stroke etiology, imprecise stratification of patients according to identified risk factors and clinical conditions, identification of other coexisting diseases, differently defined endpoints, and, finally, inadequately chosen control groups. On the other hand, failures in finding suitable ischemic stroke biomarkers can result from the heterogeneity of the cerebral stroke itself [8]. This is the consequence of pathophysiological processes occurring with different intensity in various etiopathological types of stroke, additionally complicated by different course of these processes in individual patients being the result of differences in their metabolism, age, ethnic differences, or presence of coexisting diseases. It has been postulated that the greatest chance for clinical application can have diagnostic panels composed of biomarkers characteristic for specific types of stroke [216, 218]. Biomarkers in these panels should be representative for pathophysiological processes, such as oxidative stress, necrotic and apoptotic cell death, BBB disintegration and brain edema, inflammatory response, and thrombosis. According to this approach, specificity of particular types of stroke, such as large- or small-vessel disease, lacunar stroke, and cardioembolic stroke, should be also taken into account.

An ischemic stroke is a heterogeneous process which cannot be adequately signalized by activation of a single biomarker. Therefore, it is critically important to evaluate a broad range of molecules involved in pathophysiology of stroke, to identify and choose a set of potentially the best indicators of this process [6]. Recent studies of the blood-derived 21 biomarkers containing diagnostic panel, conducted in order to differentiate between real strokes and stroke mimics and between ischemic and hemorrhagic strokes in the hyperacute phase, have failed to provide a conclusive solution, due to insufficient accuracy of differential diagnosis [219]. Therefore, searching for more effective markers of ischemic stroke still remains in the focus of interest of experts, representing different disciplines [3, 220]. Future studies will undoubtedly concentrate on isolation of substances, representing different chemical structures and creating diagnostic panels, representative for several pathophysiological processes of ischemic stroke [8, 217]. Hence, it seems justified to plan a new search for biomarkers based on several premises, resulting from the current state of knowledge of the ischemic stroke pathophysiology. Among these, the following factors should be taken into account: (1) morphological and functional specificity of cellular components of damaged tissue; (2) different resistance of various NVU components to decreased cerebral blood flow and glucose availability, enabling adaptation of metabolic processes to anaerobic conditions; (3) disintegration of neurotransmitter and signaling pathways; (4) limited sensitivity and specificity of biomarkers, resulting from their expression by several constituents of NVU and in the course of different pathological processes, such as cerebral hemorrhage, inflammation, or neurodegenerative diseases; (5) complex character of inflammatory response to ischemia; (6) simultaneous activation of several pathological processes (e.g., oxidative stress, thrombosis, brain edema, and hemorrhagic transformation), related to secretion of numerous markers; (7) dynamic character of biomarker expression, resulting in reaching their peaks at different time points after ischemia onset; (8) occurrence of various combinations of biomarkers, activated in different types of ischemic stroke (e.g., cardioembolic, lacunar, large-vessel disease); (9) patients’ age and coexistence of other diseases (e.g., cardiovascular, neurodegenerative, metabolic, inflammatory) influencing the ischemic biomarkers expression; (10) need for elaboration of complex diagnostic protocols, based on the standardized assessment of biomarker panels which should be used in combination with neuroradiological imaging studies and evaluation of gene expression and polymorphism.

In this review, we have concentrated on a selection of substances, which are representative for the components of NVU and released from the brain into blood or CSF after stroke. According to this approach, the new diagnostic panels should contain the representatives of glial proteins (e.g., S100β, GFAP, AQP4), pro- and anti-inflammatory cytokines (IL-4, IL-6, IL-10, TNFα, MMP-9), factors representing hemostatic system (vWF), markers of NVU metabolism (FABPs, NSE) and neurotransmitter synthesis (GS, SR), representatives of amino acid neurotransmitters (e.g., Glu, GABA, Gly, Ser), and, finally, neurotrophic and growth factors (BDNF, GDNF, NGF). Representation of different biochemical and functional categories by the abovementioned substances will enable monitoring of main pathophysiological processes, which occur during NVU damage. However, in several cases of the presented potential candidates for stroke biomarkers, available data related with their function are based only on experimental studies and not supported with sufficient clinical evaluation, in particular, concerning their role in various types of stroke. This justifies the need for further studies aimed at verification of their diagnostic utility.

Apart from the abovementioned functional division, another one based on the time point of expression in relation to the onset of stroke symptoms may be of practical value [8]. Assessment of ischemic stroke indicators (e.g., proinflammatory cytokines and hemostatic factors) in patients with known risk factors, prior to the onset of stroke symptoms, may have diagnostic and screening value and allow for selection of “at risk” of stroke individuals. It may also have some therapeutic value, justifying the inclusion of these patients into the group for primary prevention. Monitoring of biomarkers in the acute phase of stroke could be of great diagnostic importance. It relies on differentiation between stroke patients and the negative control individuals (biomarkers: S100β, IL-6, MMP-9, TNFα, and vWF), exclusion of stroke mimics, such as migraine, epilepsy, structural brain lesions (S100β, MMP-9), and differentiation between ischemic and hemorrhagic stroke (GFAP, S100β) [3, 216, 217]. Many of the NVU-derived markers could be used for assessment of the risk of stroke-related complications. These include hemorrhagic transformation of the ischemic stroke (S-100β, vWF, and MMP-9), development of malignant stroke (S100β), increasing infarct volume (S-100β, NSE, MMP-9, IL-6, TNFα, and Glu), early neurological deterioration and progressive (unstable) stroke or death (IL-6, S100β, TNFα, MMP-9; NSE, Glu, and GABA) [3, 217, 221]. Furthermore, there are premises suggesting value of some biomarkers in the diagnosis of specific stroke types, such as cardioembolic stroke (vWF, TNFα), hemorrhagic stroke (GFAP, MMP-9), large- and small-artery disease (IL-6; TNFα), or lacunar stroke (Glu, GABA) [3, 66, 217, 221]. Potential benefits resulting from the ischemic biomarkers’ assessment in the later phase of stroke can be related with information about its progression (biomarkers: GFAP, S100β, IL-6, MMP-9), monitoring of the results of therapy (IL-6, S100β, MMP-9), and estimation of the outcome prognosis (IL-6, S100β, vWF; BDNF) [3, 217, 221].

## Neurovascular Unit-Based Approach to Selection of New Ischemic Stroke Biomarkers

Proposed in this review approach, based on the NVU concept, for selection of potential ischemic stroke biomarkers takes into account the complexity of brain tissue and functional relationships between its cellular components. For example, the ischemic lesion affecting perivascular axonal terminals, together with the astrocytic end-foots and functional BBB, can be manifested by a changed concentration of specific biomarkers (e.g., AQP4, BDNF, GFAP, NGF, and vWF) signaling the extent and dynamics of brain damage. Similarly, disintegration of structural and functional relationships between neurons and astrocytes results in decreased level of energetic metabolism, dysfunction of tripartite synapse and impaired gliotransmission, which is signalized by release of several biomarkers (e.g., FABPs, GFAP, S100β, GABA, Glu, and NSE). Furthermore, the NVU involvement in inflammatory response initiated by ischemic process can be inferred from increased secretion of characteristic mediators (e.g., IL-4, IL-6, IL-10, MMP-9, vWF) and their transfer into the vascular system. Thus, the NVU concept proves to be useful not only for selection of potential biomarkers, representing different components of this unit, but also for determination of those of them, which are representative for pathophysiological processes occurring at different phases of stroke. In our view, another possibility for application of the NVU concept for improvement of cerebral stroke diagnosis could be related with development of methods, which combine the results of morphological studies on distribution and concentration of selected markers (e.g., AQP4, amino acids, and metabolites) with the results of neuroradiological studies, assessing the depth of cerebral blood flow disturbances, evolution of brain edema and penumbra.

## Summary and Conclusion

Ischemic stroke is a dynamic process which consequences result from the coexistence of several phenomena, such as oxidative stress, immunological response, reactive gliosis, and dysfunction of hemostatic system. In spite of continuous development of new diagnostic methods, recognition of the cerebral ischemic infarct, especially in its acute phase, is often very difficult or even impossible. Despite activation of numerous markers, their diagnostic value is not satisfactory and requires further investigation, especially in different types of stroke. This justifies the approach relying on selection of several potential markers, characterized by acceptable sensitivity and specificity, associated with acute or later phases of stroke and representative for various pathophysiological processes. Taking into account still unsatisfactory results of studies on ischemic stroke biomarkers, it may be assumed that further efforts in this area will concentrate on searching for new components of diagnostic panels representing various elements of NVU. New complex diagnostic protocols will include analysis of biomarkers’ content, neuroradiological studies, and patient’s genetic profile, including the gene polymorphism studies associated with an increased risk of stroke and its complications.
